# *In vitro* transcriptomic analyses reveal pathway perturbations, estrogenic activities, and potencies of data-poor BPA alternative chemicals

**DOI:** 10.1093/toxsci/kfac127

**Published:** 2022-12-19

**Authors:** Geronimo Matteo, Karen Leingartner, Andrea Rowan-Carroll, Matthew Meier, Andrew Williams, Marc A Beal, Matthew Gagné, Reza Farmahin, Shamika Wickramasuriya, Anthony J F Reardon, Tara Barton-Maclaren, J Christopher Corton, Carole L Yauk, Ella Atlas

**Affiliations:** Environmental Health Science and Research Bureau, Healthy Environments and Consumer Safety Branch (HECSB) Health Canada, Ottawa, Ontario K2K 0K9, Canada; Department of Biology, Faculty of Science, University of Ottawa, Ottawa, Ontario K1N 9A7, Canada; Environmental Health Science and Research Bureau, Healthy Environments and Consumer Safety Branch (HECSB) Health Canada, Ottawa, Ontario K2K 0K9, Canada; Department of Biology, Faculty of Science, University of Ottawa, Ottawa, Ontario K1N 9A7, Canada; Environmental Health Science and Research Bureau, Healthy Environments and Consumer Safety Branch (HECSB) Health Canada, Ottawa, Ontario K2K 0K9, Canada; Department of Biology, Faculty of Science, University of Ottawa, Ottawa, Ontario K1N 9A7, Canada; Environmental Health Science and Research Bureau, Healthy Environments and Consumer Safety Branch (HECSB) Health Canada, Ottawa, Ontario K2K 0K9, Canada; Department of Biology, Faculty of Science, University of Ottawa, Ottawa, Ontario K1N 9A7, Canada; Environmental Health Science and Research Bureau, Healthy Environments and Consumer Safety Branch (HECSB) Health Canada, Ottawa, Ontario K2K 0K9, Canada; Department of Biology, Faculty of Science, University of Ottawa, Ottawa, Ontario K1N 9A7, Canada; Bureau of Chemical Safety, Health Canada; Existing Substances Risk Assessment Bureau, Healthy Environments and Consumer Safety Branch, Health Canada, Ottawa, Ontario K2K 0K9, Canada; Existing Substances Risk Assessment Bureau, Healthy Environments and Consumer Safety Branch, Health Canada, Ottawa, Ontario K2K 0K9, Canada; Existing Substances Risk Assessment Bureau, Healthy Environments and Consumer Safety Branch, Health Canada, Ottawa, Ontario K2K 0K9, Canada; Existing Substances Risk Assessment Bureau, Healthy Environments and Consumer Safety Branch, Health Canada, Ottawa, Ontario K2K 0K9, Canada; Existing Substances Risk Assessment Bureau, Healthy Environments and Consumer Safety Branch, Health Canada, Ottawa, Ontario K2K 0K9, Canada; Existing Substances Risk Assessment Bureau, Healthy Environments and Consumer Safety Branch, Health Canada, Ottawa, Ontario K2K 0K9, Canada; Center for Computational Toxicology and Exposure, US Environmental Protection Agency, Research Triangle Park, North Carolina, USA; Department of Biology, Faculty of Science, University of Ottawa, Ottawa, Ontario K1N 9A7, Canada; Environmental Health Science and Research Bureau, Healthy Environments and Consumer Safety Branch (HECSB) Health Canada, Ottawa, Ontario K2K 0K9, Canada; Department of Biochemistry, Faculty of Medicine, University of Ottawa, Ottawa, Ontario K1H 8M5, Canada

**Keywords:** TempO-Seq, BPA, biomarker, benchmark concentration, potency, toxicogenomics

## Abstract

Since initial regulatory action in 2010 in Canada, bisphenol A (BPA) has been progressively replaced by structurally related alternative chemicals. Unfortunately, many of these chemicals are data-poor, limiting toxicological risk assessment. We used high-throughput transcriptomics to evaluate potential hazards and compare potencies of BPA and 15 BPA alternative chemicals in cultured breast cancer cells. MCF-7 cells were exposed to BPA and 15 alternative chemicals (0.0005–100 µM) for 48 h. TempO-Seq (BioSpyder Inc) was used to examine global transcriptomic changes and estrogen receptor alpha (ERα)-associated transcriptional changes. Benchmark concentration (BMC) analysis was conducted to identify 2 global transcriptomic points of departure: (1) the lowest pathway median gene BMC and (2) the 25th lowest rank-ordered gene BMC. ERα activation was evaluated using a published transcriptomic biomarker and an ERα-specific transcriptomic point of departure was derived. Genes fitting BMC models were subjected to upstream regulator and canonical pathway analysis in Ingenuity Pathway Analysis. Biomarker analysis identified BPA and 8 alternative chemicals as ERα active. Global and ERα transcriptomic points of departure produced highly similar potency rankings with bisphenol AF as the most potent chemical tested, followed by BPA and bisphenol C. Further, BPA and transcriptionally active alternative chemicals enriched similar gene sets associated with increased cell division and cancer-related processes. These data provide support for future read-across applications of transcriptomic profiling for risk assessment of data-poor chemicals and suggest that several BPA alternative chemicals may cause hazards at similar concentrations to BPA.

Bisphenol A (BPA) is an endocrine disruptor that interacts with nuclear hormone receptors, as well as altering non-hormonal pathways, to exert its effects (Cimmino *et al.*, 2020). Exposure to BPA is associated with multi-organ toxicity and negative health outcomes including metabolic and endocrine dysfunction, reproductive and developmental disorders, and hormone-related cancer (Ma *et al.*, 2019). Canada was the first country to take regulatory action to limit exposure to BPA in 2008, followed by the United States, the EU, and others (Rogers, 2021). As pressures to phase out BPA increase, manufacturers are relying more on BPA alternative chemicals.

BPA alternative chemicals are detected in the environment, consumer products, and in humans (Chen *et al.*, 2016). Many of these chemicals are structurally similar to BPA but differ in their adjoining atom and aryl substituents. A systematic review has highlighted the ability of bisphenol S (BPS), a sulfone-based bisphenol, and bisphenol F (4,4′-BPF), a BPA analogue missing the geminal methyl groups, to act as endocrine disruptors at similar concentrations to BPA (Rochester and Bolden, 2015). Some substitutes are less structurally related to BPA, like Pergafast201 and 4,4″-bis-(p-tolylsulfonyl ureido)-diphenylmethane (BTUM); these are non-phenolic sulfonyl urea-based alterative chemicals used primarily in thermal paper (Björnsdotter *et al.*, 2017). Several of these alternative chemicals are also associated with endocrine disruption, reproductive toxicity, and carcinogenicity (den Braver-Sewradj *et al.*, 2020). Thus, there is a clear need to identify potential hazards and determine relative potencies of BPA alternative chemicals.

High-throughput transcriptomics (HTTr) is increasingly used to identify hazards and establish bioactivity thresholds for regulatory toxicology (Thomas *et al.*, 2019). We recently applied HTTr to compare potencies of BPA alternative chemicals using benchmark concentration (BMC) analysis in H9 human embryonic stem cells exposed to BPA, BPS, 4,4′-BPF, and 3,3′,5,5′-tetrabromobisphenol A (TBBPA) at a range of concentrations (1–700 µM) (Peshdary *et al.*, 2021). BPA, BPS, and 4,4′-BPF had overlapping median BMCs (approximately 100 µM), suggesting similar potencies, whereas the BMC of TBBPA was much higher. These data must be interpreted with caution as the absence of ERs in human embryonic stem cells underestimates the toxicity of BPA and similar compounds as the primary mode action of these chemicals is via these nuclear hormone receptors.

An important approach to screening BPA alternatives is to specifically compare potencies based on interactions with the estrogen receptor (ER). For example, Kitamura *et al.* (2005) tested the effects of several BPA alternatives using MCF-7 cells transfected with an estrogen response element (ERE) luciferase reporter. Bisphenol AF (BPAF) and bisphenol B (BPB) stimulated estrogenic activity at lower concentrations than BPA, whereas BPS and 4,4′-BPF required higher concentrations. Mesnage *et al.* (2017) found that BPAF, a hexafluoro bisphenol, was the most potent BPA alternative in stimulating an ERE-mediated luciferase reporter gene relative to BPB, bisphenol Z (BPZ), BPA, 4,4′-BPF, bisphenol AP (BPAP), and BPS (Mesnage *et al.*, 2017). The same study also conducted whole genome transcriptomic analysis with MCF-7 cells and observed that these BPA alternatives perturbed Gene Ontology (GO) gene sets associated with cell cycle, steroid hormone response, and breast cancer.

Nuclear receptor activation can also be assessed through the analysis of transcriptomic biomarkers. Toward this, a 46 gene transcriptomic biomarker associated with ERα was developed and validated in ERα-positive MCF-7 cells (Ryan *et al.*, 2016). This biomarker predicts compounds that activate ERα through statistical comparisons of gene expression profiles of the 46 biomarker genes, with a pattern of 32 upregulated and 14 downregulated genes following ER activation. This approach can be used to identify the potential for the chemicals to interact with ERα and produce a composite BMC score for the biomarker to compare potencies for this specific molecular initiating event.

In this study, we explored the use of HTTr to establish mechanistic similarities, compare potencies, and increase understanding of data-poor BPA alternative chemical toxicity in support of prioritization and assessment activities by Health Canada. MCF-7 cells were exposed to BPA and 15 BPA alternatives (from 0.0005 to 100 µM) or solvent (0.1% DMSO) for 48 h. The exposure concentrations chosen span several orders of magnitude above and below the mean concentration of BPA in human urine (approximately 10 nM; Chen *et al.*, 2016) and are consistent with those used in the US Environmental Protection Agency’s ToxCast Program. HTTr using Templated Oligo-Sequencing (TempO-Seq; BioSpyder Inc) was used to examine both general toxicological transcriptional effects as well as ERα-associated transcriptional changes induced by these chemicals. BMC analysis was conducted to identify both general and ERα-specific transcriptomic points of departure (tPODs). Finally, genes showing robust concentration-response were subjected to upstream regulator and pathway analysis to explore the toxicodynamic similarity of these chemicals to each other and BPA.

## Materials and methods

###  

####  

##### Chemicals

See Table 1 for a list of chemicals and suppliers. The final concentration of DMSO in media was 0.1% for all chemicals tested.

**Table 1.** List of chemicals and suppliers

Chemical name	Abbreviation	CASRN	Purity (%)	Supplier
2,2-Bis(4-hydroxyphenyl)propane	BPA	80-05-7	>99	Sigma-Aldrich Inc (St Louis, Missouri)
Bis(4-hydroxyphenyl)methane	4,4′-BPF	620-92-8	>98	
1,1-Bis(4-hydroxyphenyl)-1-phenylethane	BPAP	1571-75-1	99	
4,4′-Sulfonyldiphenol	BPS	80-09-1	98	
2,2-Bis[4-(glycidyloxy)phenyl]propane	BADGE	1675-54-3	98	
4,4′-Dichlorodiphenyl sulfone	DDS	80-07-9	98	
4,4′-(Hexafluoroisopropylidene)diphenol	BPAF	1478-61-1	97	
Dimethyl sulfoxide	DMSO	67-68-5	>99	
17β-estradiol	E2	50-28-2	>98	
Dexamethasone	Dex	50-02-2	98	
4-[[4-(2-Propen-1-yloxy)phenyl]sulfonyl]phenol	BPS-MAE	97042-18-7	97	Toronto Research Chemicals (Toronto, ON, Canada)
4,4″-Bis-(p-tolylsulfonyl ureido)-diphenylmethane	BTUM	151882-81-4	98	
4-((4-Isopropoxyphenyl)sulfonyl)phenol	D-8	95235-30-6	98	
4-Methyl-N-[[[3-[[(4-methylphenyl)sulfonyl]oxy]phenyl]amino]carbonyl]benzenesulfonamide	Pergafast201 (P201)	232938-43-1	98	
2,4′-Dihydroxydiphenyl sulfone	2,4′-BPS	5397-34-2	98	
4-(4-Hydroxy-3-prop-2-enylphenyl)sulfonyl-2-prop-2-enylphenol	TGSA	41481-66-7	98	
2,2-Bis(4-hydroxy-3-methylphenyl)propane	BPC	79-97-0	98	Accustandard (New Haven, Connecticut)
2,4′-Dihydroxydiphenylmethane	2,4′-BPF	2467-03-0	>98	TCI America (Portland, Oregon)
4-((4-(Benzyloxy)phenyl)sulfonyl)phenol	BPS-MPE	63134-33-8	>98	

##### Cell culture

MCF-7 cells (ATCC, Manassas, Virginia) were routinely cultured in 100 mm dishes (Corning Falcon No. 353003, Corning, New York) with Dulbecco’s modified Eagle’s medium (DMEM; Gibco, Thermo Fisher Scientific, Waltham, Massachusetts) supplemented with 10% fetal bovine serum (FBS; Wisent Bioproducts, Saint-Jean Baptiste, QC, Canada), and 1% penicillin and streptomycin, at 37°C in a humidified atmosphere with 5% CO_2_. For the experiment, cells were seeded into clear 96-well plates (Corning Falcon No. 353075) at a density of 2.5 × 10^4^ cells per well in phenol red-free DMEM supplemented with 5% charcoal-dextran stripped FBS (CD-FBS) to eliminate estrogenic components (Atlas *et al.*, 2003). The next day the medium was removed and replaced with DMEM with 5% CD-FBS containing the chemicals of interest. Cells were exposed, in quadruplicate, to BPA and 15 alternative chemicals at 10 different concentrations (0.0005, 0.001, 0.01, 0.1, 0.5, 1, 5, 10, 50, 100 µM), the positive control 17β-estradiol (E2; 0.0001, 0.001, 0.01, 0.1, 1, 10 nM), dexamethasone (Dex—a non-ER-interacting control; 0.0001, 0.001, 0.01, 0.1, 1 µM) alongside solvent controls (0.1% DMSO; 4 solvent controls per plate) for 48 h. The exposure timeframe was selected based on pilot studies to ensure that we observed a robust response in estrogen-responsive genes (data not shown). After exposure, cells were washed with phosphate-buffered saline (PBS) and lysed *in situ* using 2× TempO-Seq lysis buffer diluted with an equal amount of PBS for Templated Oligo-Sequencing (TempO-Seq). We observed precipitate in some chemicals and removed these concentrations from further analysis: BPAF (50, 100 µM), BPC (50, 100 µM), BADGE (50, 100 µM), D-8 (50, 100 µM), DDS (10, 50, 100 µM), BTUM (50, 100 µM), TGSA (50, 100 µM), BPS-MPE (50, 100 µM).

##### Cell viability assay

To determine cytotoxic concentrations, MCF-7 cells were seeded into black 96-well plates (Corning Falcon No. 3603) and treated as described above. Cell viability was measured using the CellTiter-Blue Cell Viability Assay (Promega Corp, Madison, Wisconsin), as per the manufacturer’s instructions. This is a fluorometric assay that measures the metabolic capacity of cells based on resazurin reduction. Briefly, after 48-h exposure, CellTiter-Blue reagent was added to each well, and the plates were incubated at 37°C, 5% CO_2_ for 2 h. The fluorescence was read using excitation wavelength of 560 nm and emission of 590 nm using a SpectraMax M2 (Molecular Devices LLC, San Jose, California). Fluorescence readings were expressed as a % ratio to that of the DMSO control group. Cytotoxicity was defined as readings <50% of the control.

##### Cell proliferation assay

MCF-7 cells were seeded at a density of 2.0×10^4^ cells/well in clear 24-well plates (Corning Costar No. 3524) and incubated at 37°C and 5% CO_2_ in phenol red free DMEM/F12 media containing 5% CD-FBS and 1% penicillin-streptomycin. Cells were treated with BPA and 15 alternative chemicals at 11 different concentrations (0.0001–100 µM) and positive control E2 (0.0001–10 nM). Chemical concentrations that yielded precipitates were again excluded from this analysis. Cells were counted on day 7 using a TC20 automated cell counter (Bio-Rad, Hercules, California) as per manufacturer’s instructions after being treated every 2 days with indicated concentration of the bisphenols and E2.

##### TempO-Seq library building and next-generation sequencing

Gene expression was measured using the TempO-Seq Human Whole Transcriptome v2.0 kit (BioSpyder Technologies Inc, Carlsbad, California) as per manufacturer’s instructions and previously described (Yeakley *et al.*, 2017). Cell lysates and positive technical controls (Human Universal Reference RNA—uhrRNA Agilent Cat No. 740000, Santa Clara, California, and Human Brain Total RNA brRNA—ThermoFisher AM7962, Waltham, Massachusetts), as well as no-cell negative controls (1× TempO-Seq lysis buffer alone) were hybridized to the Detector Oligo (DO) Pool in an annealing kit for the whole human genome supplied by BioSpyder for 10 min at 70°C followed by a temperature gradient with a ramp rate of 0.5°C/min to 45°C over 50 min with a 16-h hold at 45°C and then cooled to 25°C. Nuclease digestion was employed to remove excess, unbound, or incorrectly bound DOs at 37°C for 90 min. Amplification templates were generated by ligating DO pairs bound to adjacent target sequences for 1 h at 37°C, followed by enzyme denaturation for 15 min at 80°C. Amplification templates (10 µL) were pipetted into a 96-well PCR Pre-Mix and Primer plate supplied by BioSpyder and amplified using a CFX96 Real-Time PCR Detection System (Bio-Rad) to incorporate a unique sample index/tag sequence and the sequencing adaptors for each sample. The following PCR settings were used: 37°C for 10 min, 95°C for 1 min; 25 cycles of 95°C for 10 s, 65°C for 30 s, 68°C for 30 s (with optical read for visual sample quality control [QC]); 68°C for 2 min; and hold at 25°C prior to library pooling and purification. For a list of attenuators used, see Supplementary File 1.

NucleoSpin Gel and PCR Clean-up kits were used to pool and purify labeled amplicons. Next-generation sequencing (NGS) libraries were sequenced using a NextSeq 500 High-Throughput Sequencing System (Illumina, San Diego, California), using 50 cycles from a 75-cycle high-throughput flow cell to achieve a median read depth of 2 million reads per sample. Data were processed as described below and reads were aligned to the BioSpyder TempO-Seq Human Whole Transcriptome probe set (22 537 probes over 19 687 genes) using their purpose-built pipeline.

##### Data processing and QC

Reads were demultiplexed from the BCL files and processed into FASTQ files using bcl2fastq v2.20.0.422. The FASTQ files were processed with the TempO-SeqR analysis pipeline in R (version 3.1) supplied by BioSpyder. This script uses STAR v2.7.8a (Dobin *et al.*, 2013) to perform alignment of raw reads to the reference sequence and the qCount function of the QuasR R package (v1.30.0; Gaidatzis *et al.*, 2015) to extract the feature counts from the aligned reads (BAM files) using features specified in a GTF file. The script generates a samples-by-probes count matrix.

Study-wide QC was performed on the count matrix using several methods to measure consistency and remove low-quality samples, using the methods in Harrill *et al.* (2021) as a guideline. A cutoff of 0.1 for 1-Spearman’s ρ was used to remove samples that were not correlated with others in the study. As in Harrill *et al.* (2021), we also used a 10% cut-off of uniquely mapped reads as the number of target sequences (eg, 100 000 reads to pass filter when the target is 1 000 000 for TempO-Seq experiments). We removed any samples outside of Tukey’s Outer Fence (3× interquartile range) for: (1) the number of probes capturing the top 80% of the signal, and (2) the number of detected probes (those with at least 5 mapped reads). Samples with a Gini coefficient (which measures inequality in distributions) greater than 0.95 were excluded. Samples removed by these criteria are listed in Supplementary File 2. We note that a technical error led to loss of the 0.001 and 0.01 µM concentrations of BPA-exposed cells (see Supplementary Table 2).

To create a matrix for biomarker analysis, individual pairwise contrasts for each concentration and each chemical tested were created to the respective 0.1% DMSO control samples for each plate. Following the recommendations set out by the Omics Data Analysis Frameworks for Regulatory application (R-ODAF) guidelines (Verheijen *et al.*, 2022), genes were filtered for each contrast tested to include only those where 75% of at least one experimental group were above 0.5 counts per million (CPM), and spurious spikes were removed in which [max–median] of counts were less than [sum of counts]/[number of replicates+1]. We used DESeq2 1.30.0 (Love *et al.*, 2014) to estimate fold changes and normalize for library size within the TempO-Seq data. The ashr method was used to perform log2FoldChange shrinkage (Stephens, 2017). The code used to perform processing of high-throughput sequencing data is available at https://github.com/R-ODAF (last accessed December 2022).

##### Gene expression biomarker analysis

To determine if the BPA alternatives activated ER or stress pathways, the expression profile of each chemical-concentration tested relative to solvent control (derived from the analysis of biosets) was compared with a number of characterized biomarkers as previously described (Kupershmidt *et al.*, 2010). Biosets are gene expression profiles of genes filtered by unadjusted *p* value <.05 and absolute fold change ≥1.2. The biomarkers included those that predict modulation of ER (Ryan *et al.*, 2016), Nrf2 (Rooney *et al.*, 2020), heat shock factor 1 (Cervantes and Corton, 2021), metal-induced transcription factor 1 (Jackson *et al.*, 2020), TGx-DDI (Li *et al.*, 2019), TGx-HDACi (Cho *et al.*, 2021), and NF-kB (Korunes *et al.*, 2022). The correlations between each biomarker and the gene lists of BPA and alternatives were determined using the Running Fisher algorithm as described previously in the BaseSpace Correlation Engine (Ryan *et al.*, 2016). The Running Fisher algorithm provides an assessment of the statistical significance of the correlation of the overlapping genes between the biomarker and each gene list providing a summary *p* value. A complete description of the Running Fisher test is provided in Kupershmidt *et al.* (2010). The results were exported and each *p* value was converted to a −log(*p* value). Negative values were used to indicate negative correlation between the biomarker and the gene list. Thresholds for significance were set at −log(*p* value) ≥4 for activation or ≤−4 for suppression based on prior studies.

##### Points of departure analysis

We prefiltered probes using a Williams trend test (*p* < .05) and absolute fold change >1.5. Probes with the following criteria were removed: (1) having a BMC greater than the concentration used in the analysis after excluding cytotoxic/precipitating concentrations; (2) mapping to more than one gene; (3) having a model fit *p* value <.1 determined by a likelihood ratio test; and (4) having a BMC upper (BMCU) to BMC lower (BMCL) ratio <40.

BMDExpress2.3 was used to derive BMCs for BPA alternative chemicals as previously described (Farmahin *et al.*, 2017). Concentration-response modeling was done by fitting genes best fit models, including polynomial 2°, linear, power (power term constrained to ≥1), and exponential models (degrees 2–5); best fit models were selected for each probe based on the lowest Akaike Information Criterion (AIC). The BMDExpress parameters were: 250 maximum iterations, 0.95 confidence level, assume constant variance, BMR type SD, BMR factor 1 SD, restrict power ≥1. The BMDExpress model selection criteria were: compute and utilize in best model selection, best poly model test Nested Chi Square, *p* value cut-off .05. Probes that met all the BMC filtering criteria were converted to their corresponding Entrez Identifiers. Data were further analyzed using R-4.0.5 statistical software. Gene accumulation plots were made by plotting the BMC of each gene on the horizontal axis and representing the gene accumulation number along the vertical axis.

Two approaches were used for tPOD generation intended for: (1) general toxicity; and (2) ERα-specific response.

To establish general toxicity tPODs 2 methods were used:

- Lowest median pathway BMC: We produced the lowest median pathway BMC by aligning genes and their associated BMC/BMCL/BMCU values to the gene sets in the Reactome Pathways database (https://reactome.org/; last accessed December 2022), the Kyoto Encyclopedia of Genes and Genomes (KEGG; https://www.genome.jp/kegg/; last accessed December 2022), or the GO database (http://geneontology.org; last accessed December 2022). Gene sets that contained at least 3 gene BMCs (genes that pass all criteria in the analysis) and were at least 5% populated (based on total annotated gene number) were selected. The tPOD chosen represents the gene set from the pathway database with the lowest median BMC. This approach was based on one recommended by the National Toxicology Program (NTP) (National Toxicology Program, 2018).
- We set the 25th rank-ordered gene as a threshold to identify the concentration at which a robust change in the transcriptome has occurred (eg, a concerted molecular response; Johnson *et al.*, 2022). Chemicals that do not have at least 25 genes fitting BMCs were considered “inactive” based on analysis of this tPOD.

To derive an ERα-specific tPOD, we calculated the median BMC of the 46 genes from the published ERα biomarker (Ryan *et al.*, 2016). Confidence Intervals for gene sets were estimated using a parametric bootstrap. For each gene in the gene set, a uniform distribution was assumed with the lower limit represented by the gene BMCL and the upper limit represented by the gene BMCU. A bootstrapped sample consisted of randomly generated BMCs for each gene that had a BMC. The median value from each of the 2000 bootstrap samples was used to estimate the bootstrap distribution for the median. The 95% confidence interval was estimated using the 2.5th and 97.5th percentiles from the bootstrap distribution.

##### Pathway and upstream regulator enrichment analysis

Ingenuity Pathway Analysis (IPA; QIAGEN, Redwood City, California) was used to identify perturbed upstream regulators, canonical pathways, and disease & functions. For each chemical tested, we imported Excel files into IPA containing gene IDs (Ensembl and Gene Symbol) for the genes that fit a BMC (ie, passed all filtering criteria), as well as their Williams trend test *p* values from the BMC analysis, and the largest fold-changes of the gene relative to solvent controls (exported data from BMDExpress). This approach allowed us to identify enrichment of genes showing robust concentration-responses to the exposures. IPA Core Analysis with a gene expression threshold of absolute fold change ≥1.5 and FDR-adjusted *p* value ≤.05 was used with the direct and indirect relationship settings based on experimental and highly predicted data (focusing on human sources from breast cancer cell lines). Statistical significance of the overlap (FDR-adjusted *p* value ≤.05) between the data set and known targets of upstream regulators in IPA were calculated using Fisher’s exact tests. The *z*-score was calculated using Fisher’s exact test based on the expected relationship for directions between upstream regulators and target genes and those observed in the data set. A *z*-score of >2 (activated) or <2 (inhibited) was considered statistically significant.

## Results

###  

#### Cell viability assay

Cytotoxicity was evaluated using the CellTiter-Blue Cell Viability assay. There was no evidence of cytotoxicity using this assay, as there was no decline in fluorescence for BPA or any of the alternative chemicals at any of the concentrations tested following 48-h exposure (Supplementary Figure 1).

#### Cell proliferation assay

Cell proliferation was evaluated by counting cells following exposure to chemicals for 7 days and comparing with respective solvent controls. BPA (50, 100 µM) and several alternative chemicals decreased cell counts below 50% of solvent control, including: 4,4′-BPF (100 µM), BPAP (10, 50, 100 µM), BPS-MAE (50, 100 µM), 2,4′-BPF (100 µM) which suggests cell cytotoxicity (Supplementary Figure 2).

#### General HTTr data summary

Transcriptomic data were processed using the R-ODAF pipeline that assesses quality of preprocessed data, implements study-wide alignment QCs (Harrill *et al.*, 2021), and produces fold changes and *p* values for each gene with the DESeq2 R package (Love *et al.*, 2014). The median read depth was approximately 2 million per sample with a median range of 96.6% mapped reads. Of 960 samples, 59 were lost to quality assurance (QA)/QC analysis. All QC reports are available in Supplementary File 2. The data were further filtered at the gene or probe level to produce a read count matrix for analysis in BMDExpress v2.3 to derive lists of concentration-responsive genes for enrichment analysis and tPOD derivation for toxicological potency ranking.

#### Stress response biomarker activation

We used previously published transcriptomic biomarkers to explore general stress responses and toxicities following exposure to BPA and its substitutes (Supplementary File 3). The biomarkers were specific to: (1) nuclear factor erythroid 2-related factor 2 (Nrf2) activation/suppression (Rooney *et al.*, 2020); (2) DNA damage induction (TGx-DDI; Li *et al.*, 2019); (3) metal responsive transcription factor 1 (MTF1) activation/suppression (Jackson *et al.*, 2020); (4) nuclear factor kappa beta (NFκB) activation/suppression (Korunes *et al.*, 2022) and an unpublished biomarker gene set (5) for activation of heat shock transcription factor 1 (HSF1; Cervantes and Corton, 2021). We also investigated an epigenotoxicity biomarker for histone deacetylase inhibition (HDACi; Cho *et al.*, 2021). Coactivation of these biomarkers within a condition generally occurs when there are broad, non-specific changes in transcription that result from overt cellular stress; ie, the cytotoxic burst phenomenon that occurs at concentrations close to cell death (Escher *et al.*, 2020). Supplementary Table 1 provides a summary of all stress response biomarkers activated and inhibited.

BPAP activated most of the stress response biomarkers at the top 2 concentrations: Nrf2 (50, 100 µM); TGx-DDI (100 µM); MTF1 (50, 100 µM); NFkB (100 µM); and HSF1 (50, 100 µM). BPA activated both Nrf2 and MTF1 at 100 µM. 2,4′-BPF activated Nrf2 (50, 100 µM) and NFkB (50 µM). BPS activated NFkB (100 µM) and MTF (0.001 µM). BPS-MAE activated Nrf2 and MTF1 at 100 µM. P201 activated MTF1 (0.01, 0.1, 1, 10, 100 µM) and NFkB (100 µM). A few other chemicals activated a single biomarker: 4,4′-BPF activated the Nrf2 biomarker at 100 µM; 2,4′-BPS activated NFkB at 100 µM; and BADGE (0.1, 0.5, 1 µM) and BTUM (5 µM) activated MTF1. The HDACi biomarker was inhibited by BPA (5–50 µM) and the following chemicals: 4,4′-BPF (5–100 µM), BPAF (1 µM), BPAP (5–50 µM), BPC (5–10 µM), and BPS (5 µM).

#### Final sample inclusion and exclusion for ERα activity, potency comparison, and enrichment analysis

The stress response biomarker analysis was used to identify those concentrations of the chemicals that were causing overt cellular stress. Specifically, a chemical concentration was considered cytotoxic (overly stressed) if it activated 2 or more biomarkers or if it decreased cell counts below 50% of controls; these concentrations were subsequently removed from further analyses. In general, the highest exposure concentrations of BPA (50, 100 µM) and several BPA alternative chemicals met these criteria, including: 2,4′-BPF (50, 100 µM), 4,4′-BPF (100 µM) BPAP (10, 50, 100 µM), BPS-MAE (50, 100 µM), and P201 (100 µM). Supplementary Table 2 provides a summary of the final concentrations retained for: (1) ERα biomarker analysis; (2) BMC analysis; (3) tPOD derivation; (4) UR and pathway analysis. For some chemicals like BPA and BPAP, stress response biomarkers were activated at highest concentrations, whereas ERα biomarker activation at these concentrations decreased. These data support that the cellular generalized stress response was associated with a loss of ERα signaling. Further, this stress response is in line with a cytotoxic “burst” phenomenon associated with exposure to high concentrations of certain chemicals (Judson *et al.*, 2016).

#### Identification of ERα receptor agonists and potency ranking for ERα effects

**Figure 1.**
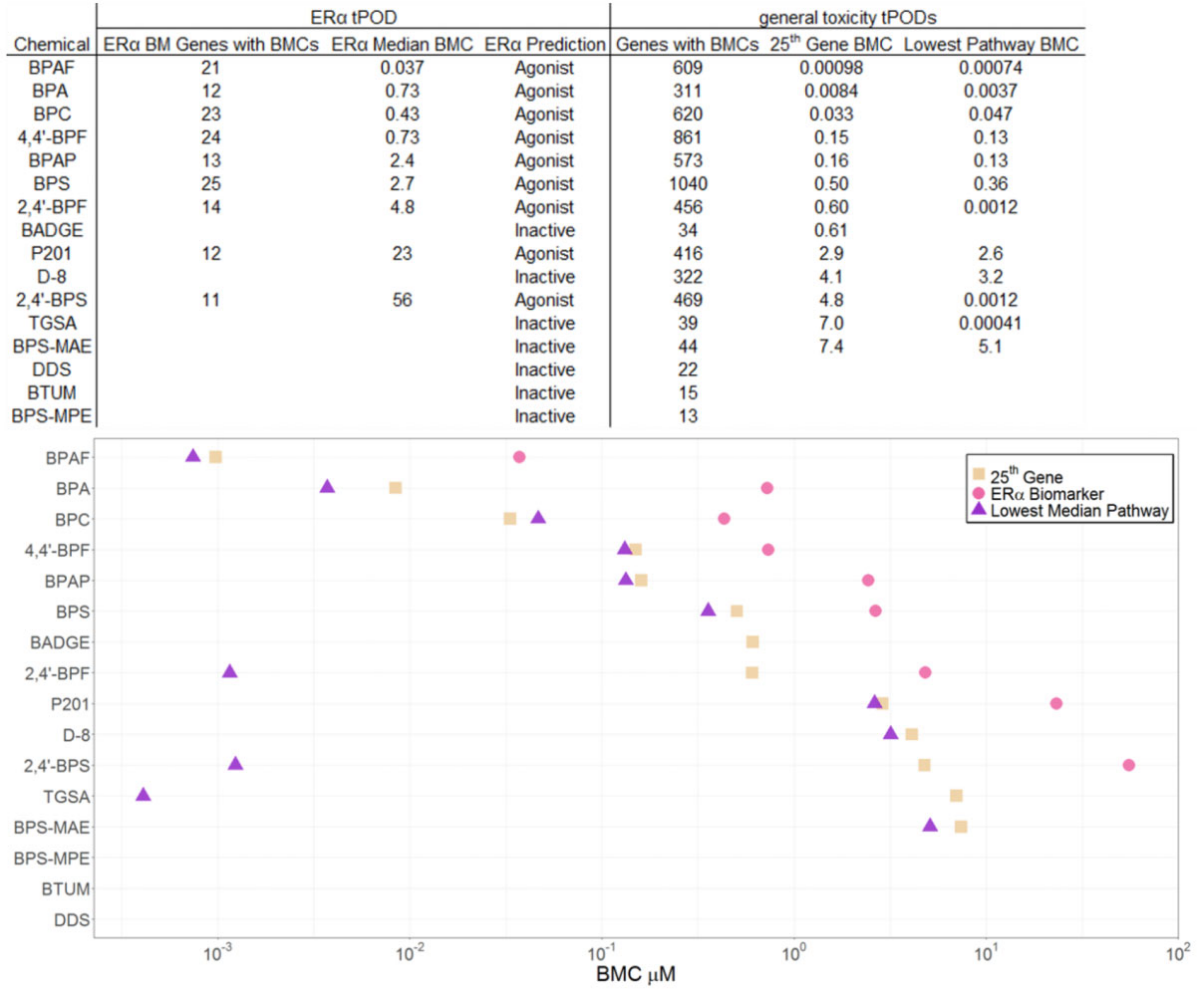
Comparison of estrogen receptor alpha (ERα) bioactivity and transcriptomic points of departure (tPODs) derived from the whole transcriptome analysis in MCF-7 cells (*n* = 3–4 per concentration) exposed to bisphenol A and 15 alternative chemicals at a range of concentrations (0.0005–100 µM) for 48 h. ERα prediction is based on at least one concentration yielding an agonist or antagonist call. For tPOD derivation, data were prefiltered using the Williams trend test (*p* ≤ .05) and a fold-change of ≥1.5 or ≤1.5. Data were also postfiltered with the following settings in BMDExpress v2.3: Best BMD/BMDL <20, Best BMDU/BMD <20, Best BMDU/BMDL <40, and Best fitPvalue >.1. tPODs representing the 25th rank ordered gene benchmark concentration (BMC) (shown in µM), the median gene BMC for the lowest pathway (at least 3 genes and 5% of pathway) as well as the median gene BMC for the ERα biomarker gene set are shown in the table and in the top panel. The chemicals are shown in decreasing order of potency based on tPODS from the 25th gene BMC.

The ability of each BPA alternative chemical tested to activate the ERα was predicted using a published 46 gene ERα biomarker (Ryan *et al.*, 2016). Nine chemicals including BPA were predicted to be ERα agonists (Figure 1 and Supplementary File 3).

The median BMCs of genes that fit models using BMDExpress from the ERα biomarker were also used to rank order the potency of these chemicals for ERα activity. The rank from most potent to least potent based on this was: BPAF, BPC, BPA, 4,4′-BPF, BPAP, BPS, 2,4′-BPF, P201, 2,4′-BPS (Figure 1).

#### Potency ranking using BMC analysis

The total number of genes fitting BMC models is shown in Figure 1. The top 2 most transcriptionally active by this analysis (ie, chemicals with the most genes fitting BMC models) were BPS and 4,4′-BPF, with 1040 and 861 genes fitting models, respectively. In contrast, 3 BPA alternative chemicals (DDS, BTUM, BPS-MPE) had fewer than 25 genes fitting BMC models (presented in more detail below).

To rank order BPA alternative chemicals by toxicological potency, we calculated 2 general systemic tPODs to represent a “tipping point” for induction of transcriptomic perturbations: (1) the 25th rank-ordered gene BMC, and (2) the lowest pathway gene set median BMC as per the U.S. NTP’s recommendation (National Toxicology Program, 2018).

**Figure 2.**
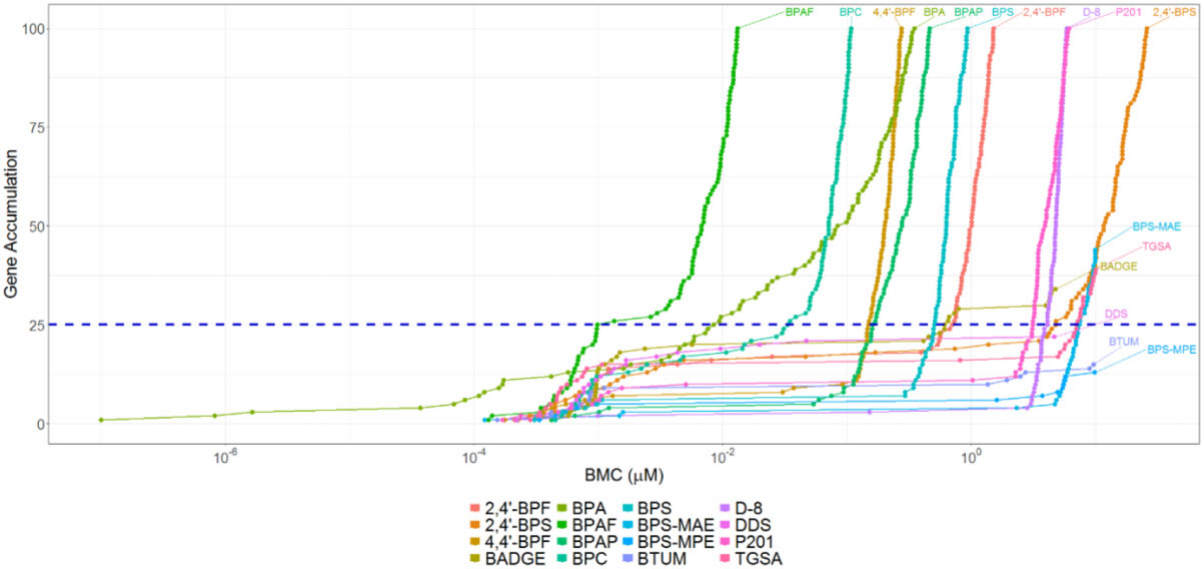
Comparison of ERα bioactivity and tPODs derived from the whole transcriptome analysis in MCF-7 cells (*n* = 3–4 per concentration) exposed to BPA and 15 alternative chemicals at a range of concentrations (0.0005–100 μM) for 48 h. ERα prediction is based on at least one concentration yielding an agonist or antagonist call. For tPOD derivation, data were prefiltered using the Williams trend test (*p* ≤ .05) and a fold-change of ≥1.5 or ≤1.5. Data were also post-filtered with the following settings in BMDExpress v2.3: Best BMD/BMDL <20, Best BMDU/BMD <20, Best BMDU/BMDL <40, and Best fitPvalue >.1. tPODs representing the 25th rank ordered gene BMC (shown in μM), the median gene BMC for the lowest pathway (at least 3 genes and 5% of pathway) as well as the median gene BMC for the ERα biomarker gene set are shown in the table and in the top panel. The chemicals are shown in decreasing order of potency based on tPODS from the 25th gene BMC. 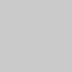 25th gene, 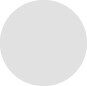 ERα biomarker, 
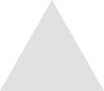
 lowest median pathway.

Gene accumulation plot shows the ranking of BPA and its alternatives potency according to their 25th ranked-ordered gene BMC (Figure 2). The order of potency from the most to least potent (lowest BMC to highest BMC) was: BPAF, BPA, BPC, 4,4′-BPF, BPAP, BPS, 2,4′-BPF, BADGE, P201, D-8, 2,4′-BPS, TGSA, BPS-MAE. Three BPA alternative chemicals (DDS, BTUM, BPS-MPE) did not have at least 25 genes with modeled BMC and thus were considered “not transcriptionally active” at the concentrations tested (Figure 1). General system tPODs were used to compare potency with our prototype agent: BPA. The halogenated bisphenol BPAF was more potent than BPA (BMC = 0.00098 µM and 0.0084 µM for BPAF and BPA, respectively). BPA is more potent than BPC (BMC = 0.033 µM) by one order of magnitude, followed by 5 alternative chemicals (4,4′-BPF, BPAP, BPS, 2,4′-BPF, BADGE) that had BMCs greater than one order magnitude higher (0.15–0.61 µM) compared with BPC. Importantly, the 25th rank-ordered gene BMC of BPA is within one or order of magnitude of the mean human urinary BPA concentration (Chen *et al.*, 2016). The remaining 5 (ie, least potent) transcriptionally active BPA alternative chemicals (P201, D-8, 2,4′-BPS, TGSA, BPS-MAE) had BMCs greater than the previous 5 compounds by one order of magnitude (2.9–7.4 µM).

We then ranked BPA and its alternatives by potency based on the lowest median BMC derived from pathways (Figure 1). The order of potency from most to least potent was: TGSA (GO), BPAF (GO), 2,4′-BPF (Reactome), 2,4′-BPS (KEGG), BPA (GO), BPC (GO), 4,4′-BPF (Reactome), BPAP (GO), BPS (GO), P201 (Reactome), D-8 (GO), BPS-MAE (GO). A full list of gene sets for each chemical tested is included in Supplementary File 4.

#### Pathway and upstream regulator enrichment analysis

**Figure 3.**
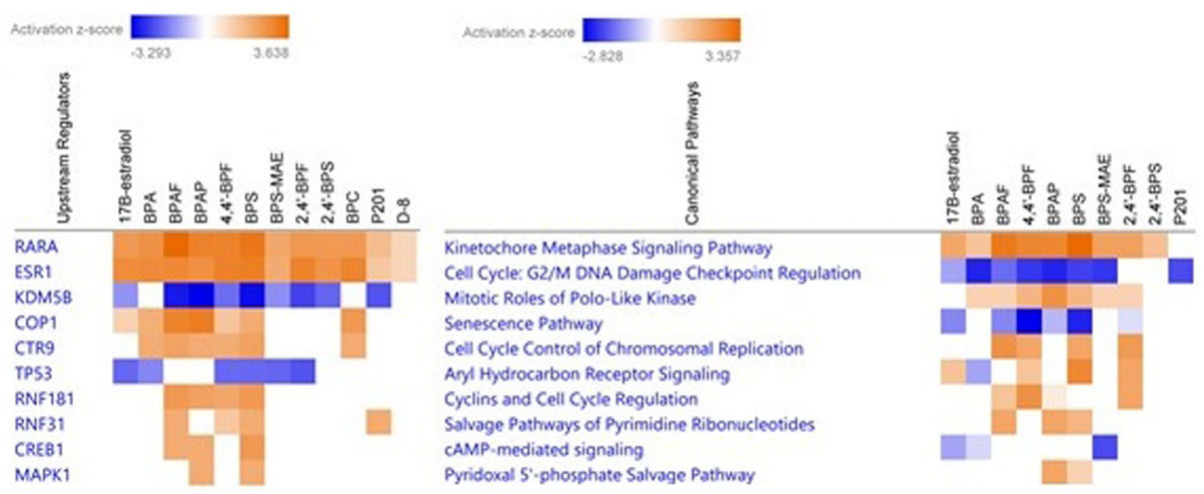
Ingenuity Pathway Analysis of genes fitting benchmark concentration models in the MCF-7 cells (*n* = 3–4 per concentration) exposed to bisphenol A and 15 alternative chemicals for 48 h, as well as positive control 17β-estradiol. A list of genes fitting models, the Williams trend test *p* value and the maximum fold-change were imported into IPA for this comparison. *Z*-score and adjusted *p* value filters were set to ≥2.0 and ≤.05, respectively. A, Upstream regulators. B, Top 10 canonical pathways.

We uploaded the lists of genes with BMCs, along with the maximum fold change and Williams trend test *p* values, into IPA to determine if there was enrichment of genes fitting models to specific canonical pathways and/or diseases, or associated with regulation by specific upstream molecules. This approach allowed us to compare chemicals across all concentrations tested and revealed that the positive control (E2), BPA, and 10 BPA alternative chemicals (BPAF, BPC, 4,4′-BPF, BPAP, BPS, BPS-MAE, 2,4′-BPF, 2,4′-BPS, BPC, P201, and D-8) perturbed gene sets associated with predicted upstream regulators and canonical pathways (Figure 3). A full list of upstream regulators, canonical pathways, and disease and functions is in Supplementary File 5. The gene set enrichment analyses were remarkably similar across the ERα active chemicals and E2, which indicates a high degree of concordance in the transcriptional alterations that they induce.

Upstream regulator analysis (Figure 3) provides additional support to the weight of evidence that many of the BPA alternative chemicals are ERα activators (see Figure 3) operating through similar mechanisms. As expected, the estrogen receptor 1 (ERα) was predicted to be activated by E2 and all the ERα agonists identified in the biomarker analysis. Further, E2 and many of the BPA alternative chemicals were also predicted to activate the retinoic acid receptor alpha (RARα). From this analysis, D-8 and BPS-MAE were predicted to activate ESR1 (ie, ERα) and RARα, albeit weakly, although these chemicals were not predicted to be ERα active using the biomarker. E3 ubiquitin ligase constitutive photomorphogenic 1 (COP1) and Paf1/RNA polymerase II complex component (CTR9) were predicted to be activated only by BPA, BPAF, 4,4′-BPF, BPAP, and BPS and ring finger protein (RNF181) activation was predicted for BPAF, 4,4′-BPF, BPAP, and BPS. The 2 upstream regulators most inhibited by E2 and many of the BPA alternative chemicals were: lysine demethylase 5B (KDM5B) and the tumor protein 53 (TP53). Overall, BPA, BPAF, 4,4′-BPF, BPAP, and BPS perturbed the most similar upstream regulators.

In general, the most affected canonical pathways were associated with sustained proliferative signaling and downregulation of growth suppressors (Figure 3). For example, E2 and most ERα active chemicals tested were predicted to activate the kinetochore metaphase signaling pathway and inhibit the cell cycle and DNA damage checkpoint control regulation pathway. Some perturbed pathways, including aryl hydrocarbon receptor and salvage pathways of pyrimidines ribonucleotides, likely represent a non-specific transcriptomic response to xenobiotic exposure. Full interpretation of altered canonical pathways is beyond the scope of this study but serves to group BPA alternative chemicals based on possible mode of action. In sum, most ERα active BPA alternative chemicals, including BPA, BPAF, 4,4′-BPF, BPAP, BPS, and 2,4′-BPF perturbed similar canonical pathways.

## Discussion

A major goal of this study was to provide information on the mode of action and potency of data-poor BPA alternatives relative to more well-characterized replacements. We used a previously published transcriptomic ERα biomarker (Ryan *et al.*, 2016) to identify ERα active compounds and derive a tPOD for potency comparison. The biomarker analysis identified 9 agonists among the 16 chemicals tested, in line with previous data. Indeed, several BPA alternative chemicals have been identified as ERα active using other methods, including BPC, 4,4′-BPF, BPS, BPAF, and BPAP (see Keminer *et al.*, 2020; Pelch *et al.*, 2019). BPAF had the lowest ERα median BMC, followed by BPC. BPA was the third most estrogenic compound tested, and had comparable estrogenicity to 4,4′-BPF, BPAP, BPS, and 2,4′-BPF. Importantly, our data align with Mesnage *et al.* (2017) who also applied this biomarker in MCF-7 cells to identify the following BPA replacements with ERα activity: BPAF, 4,4′-BPF, BPS, and BPAP. Moreover, BPAF was identified as the most potent chemical tested among 20 BPA alternatives using ToxCast data for gene sets associated with nuclear hormone receptors, including the ERα (Pelch *et al.*, 2017). Our data add to the literature base to support that BPAF is more estrogenic than BPA and other alternative chemicals *in vitro* indicating that more information is required to scrutinize BPAF as an alternative to BPA. This information could serve to support read-across chemical safety evaluations.

Several data-poor BPA replacements demonstrated weak or no estrogenicity based on our biomarker analysis. Specifically, 2,4′-BPF was less estrogenic than its isomer (4,4′-BPF) by one order of magnitude. To our knowledge, we are the first group to report on the estrogenicity of 2,4′-BPF in MCF-7 cells. Previous work suggests that P201 is non-estrogenic (Goldinger *et al.*, 2015; Keminer *et al.*, 2020); our results suggest that this alternative chemical is weakly estrogenic and ERα agonism occurs at high concentrations (≥50 µM). 2,4′-BPS was previously predicted to be ERα inactive (Keminer *et al.*, 2020; Pelch *et al.*, 2019); herein, we predicted ERα activity at high concentrations (≥50 µM). Other data-poor BPA alternatives, including TGSA, D-8, BPS-MAE, and BPS-MPE were predicted to be ERα inactive, in line with previous data (Goldinger *et al.*, 2015; Keminer *et al.*, 2020; Kuruto-Niwa *et al.*, 2005; Pelch *et al.*, 2019). We were unable to find other published reports on the estrogenicity of DDS or BTUM. Our data indicate that further investigations using established methods (eg, competitive binding assays) are warranted to confirm the estrogenic activity of 2,4′-BPF and P201.

We explored 2 approaches to identify a “general toxicological response tPOD” that reflects a concentration at which the transcriptome is perturbed based on (1) the 25th ranked gene BMC or (2) the lowest median pathway BMC. We previously used the 5th percentile as a tPOD for deriving bioactivity exposure ratios to be conservative (Rowan-Carroll *et al.*, 2021); we note that percentile gene BMCs are influenced by the top concentration and total number of responsive genes. The 5th percentile can also be derived from very few genes and we sought to identify a method that ensures some minimal level of transcriptional change. Thus, the 25th rank-ordered gene was used for the first time as a fixed point that represents a robust transcriptomic change relative to matched solvent controls (Reardon *et al.*, 2021); the 25th gene is a more stable point for direct comparisons across chemicals that is less influenced by inclusion/exclusion of top concentrations relative to the 5th percentile BMC. This was an arbitrary but conservative selection based on our previous analysis that such an approach provides reproducible potency rankings for per- and poly-fluoroalkyated chemicals and represents approximately 0.1% of the genes in the genome. The lowest median pathway was included because it is also less influenced by inclusion/exclusion of top concentrations and has previously been proposed as an acceptable approach by a panel of experts (National Toxicology Program, 2018).

The general toxicological response tPODs yielded potency rankings that largely aligned with the ERα biomarker approach. In particular, the 25th gene BMC yielded very similar potency rankings as the ERα BMC, but was generally more conservative by one order of magnitude. This suggests that there is biological activity impacting genes not directly regulated by ERα at lower exposure concentrations than those regulated by the receptor. BPAF had the lowest 25th gene BMC, followed by BPA, BPC; all other chemicals had 25th gene BMCs within 2 orders of magnitude of BPA. The lowest pathway median BMC was also very similar to the 25th gene BMC in most cases. Examination of the accumulation plots indicates the 25th gene BMC serves as a suitable representation of the concentration at which transcriptional activity ramps up. Interestingly, TGSA, 2,4′-BPF, and 2,4′-BPS had very low pathway median BMCs, whereas they were among the least potent chemicals tested according to the other 2 tPODs. This may represent a limitation to the NTP approach, which is not encountered using the 25th gene BMC. Further investigation is warranted into the transcriptomic effects of data-poor BPA alternative chemicals. Overall, these data align with previous data from our group that found considerable overlap between pathway- and global-based tPODs from microarray data of *in vivo* toxicological dose-response studies (Farmahin *et al.*, 2017).

We used a suite of previously published transcriptomic biomarkers to explore stress responses induced by the chemicals in the MCF-7 cells (Jackson *et al.*, 2020; Korunes *et al.*, 2022). Most cellular stress response biomarkers, as well as the DNA damage biomarker, were activated by BPA and alternative chemicals at the highest (≥50 µM) concentrations tested. We also used a novel HSF1 biomarker to assess cellular stress and observed a similar trend. Interestingly, BPA and several alternatives inhibited the HDACi biomarker at non-cytotoxic concentrations. Thus, these BPA alternatives may decrease histone acetylation, perhaps indirectly, adding to a growing body of evidence that supports that *in vitro* and *in vivo* BPA exposure affects this pathway (Qin *et al.*, 2020). Overall, those alternative chemicals with the most structural homology to BPA had the most similar patterns of biomarker perturbation.

Genes fitting BMCs revealed toxicodynamic similarities across the chemical sets supporting read-across for data-poor BPA alternative chemicals. All chemicals identified as ERα active by biomarker analysis were also predicted to activate ERα as an upstream regulator, along with RARα. RARα is necessary for ERE-mediated transcription and proliferation in breast cancer cells (Ross-Innes *et al.*, 2010) and has significantly overlapping target genes with ERα (Hua *et al.*, 2009). Most ERα active BPA alternative chemicals were predicted to inhibit KDM5B. Previous work supports a role for KDM5B in cultured breast cancer cell proliferation (Zhang *et al.*, 2019), and its predicted inhibition in our dataset may represent a compensatory mechanism in response to sustained proliferative signaling by BPA alternative chemicals. Furthermore, a subset of the ERα active chemicals activated upstream regulators that facilitate ERα signaling including CTR9 (Zeng *et al.*, 2016), RNF31 (Zhu *et al.*, 2014), RNF181 (Zhu *et al.*, 2020), and COP1 (Tang *et al.*, 2022). These data support our identification of ERα active BPA alternatives and suggest that they cause robust activation of the ERα proliferative network in MCF-7 cells.

Our pathway analysis suggests that several BPA alternatives induce transcriptional changes in gene sets associated with non-specific proliferative signaling and dysregulated cell cycle control. MAPK1 and CREB1 were predicted as activated upstream regulators by a subset of chemicals tested. Another group identified CREB1 as an upstream regulator in hippocampi of BPA-exposed mice using RNA-seq (Henriksen *et al.*, 2020). Further, the most perturbed canonical pathways in this study by BPA and alternative chemicals were the kinetochore metaphase signaling pathway, mitotic roles of polo-like kinase, and G2/M DNA damage checkpoint. Importantly, a previous transcriptomic profiling study using RNA-seq in MCF-7 cells found that BPA and 5 alternative chemicals induced GO pathways associated with cell cycle and breast cancer (Mesnage *et al.*, 2017). Collectively, these enriched gene set data suggest that exposure to BPA and several alternative chemicals affect the ability of MCF-7 cells to regulate cell division.

In conclusion, our HTTr data analysis pipeline enabled us to identify potential ERα agonists within our 15 BPA alternative chemicals, several of which are supported by additional evidence in the literature. Application of BMC modeling facilitated toxicological potency ranking based both on ER-specific hazard as well as more generalized transcriptomic responses. A novel approach to gene set enrichment based on genes fitting BMC models revealed a high degree of similarity across the gene expression profiles induced by these chemicals, supporting their grouping for this and future analyses. This study also supports previous data indicating that BPAF is a potent ERα agonist that induces transcriptional changes at low concentrations, indicating that further research of halogenated BPA alternative chemicals is warranted. Given that these data are derived from an *in vitro* cell model that overexpresses the ERα, they are limited in their ability to generalize to human toxicity. Thus, further investigation in primary cell models would be beneficial. Together, our study provides key support for future read-across applications and suggests that several of the proposed BPA alternative chemicals may cause hazards at similar concentrations to BPA.

## Supplementary Material

kfac127_Supplementary_Data
