# Supplementary material for: In vitro transcriptomic analyses reveal pathway perturbations, estrogenic activities, and potencies of data-poor BPA alternative chemicals
Source: Toxicol Sci. 2022 Dec 19;191(2):266–75. doi: 10.1093/toxsci/kfac127 (PMC9936204; doi:10.1093/toxsci/kfac127)
Supplement: kfac127_Supplementary_Data [file kfac127_supplementary_data.zip › kfac127_Supplementary_Data/toxsci-22-0281-File002.docx]

Supplementary Materials

*In vitro* transcriptomic analyses reveal pathway perturbations, estrogenic activities, and potencies of data-poor BPA alternative chemicals

**Authors**: Matteo, G^1,2^, Leingartner K^2^, Rowan-Carroll A^2^, Meier M^2^, Williams A^2^, Beal MA^3^, Gagné M^4^, Farmahin R^4^, Wickramasuriya S^4^, Reardon AJF^4^, Barton-Maclaren T^4^, Corton J, Christopher^5^, Yauk CL^1,2*^, Atlas E^2,6*^.

^1^Dept. of Biology, University of Ottawa

^2^Environmental Health Science and Research Bureau, Health Canada

^3^Bureau of Chemical Safety, Health Canada

^4^Existing Substances Risk Assessment Bureau, Health Canada

^5^Center for Computational Toxicology and Exposure, US Environmental Protection Agency

^6^Dept. of Biochemistry, University of Ottawa.

* To whom correspondence should be addressed. E-mail: ella.atlas@hc-sc.gc.ca and carole.yauk@uottawa.ca


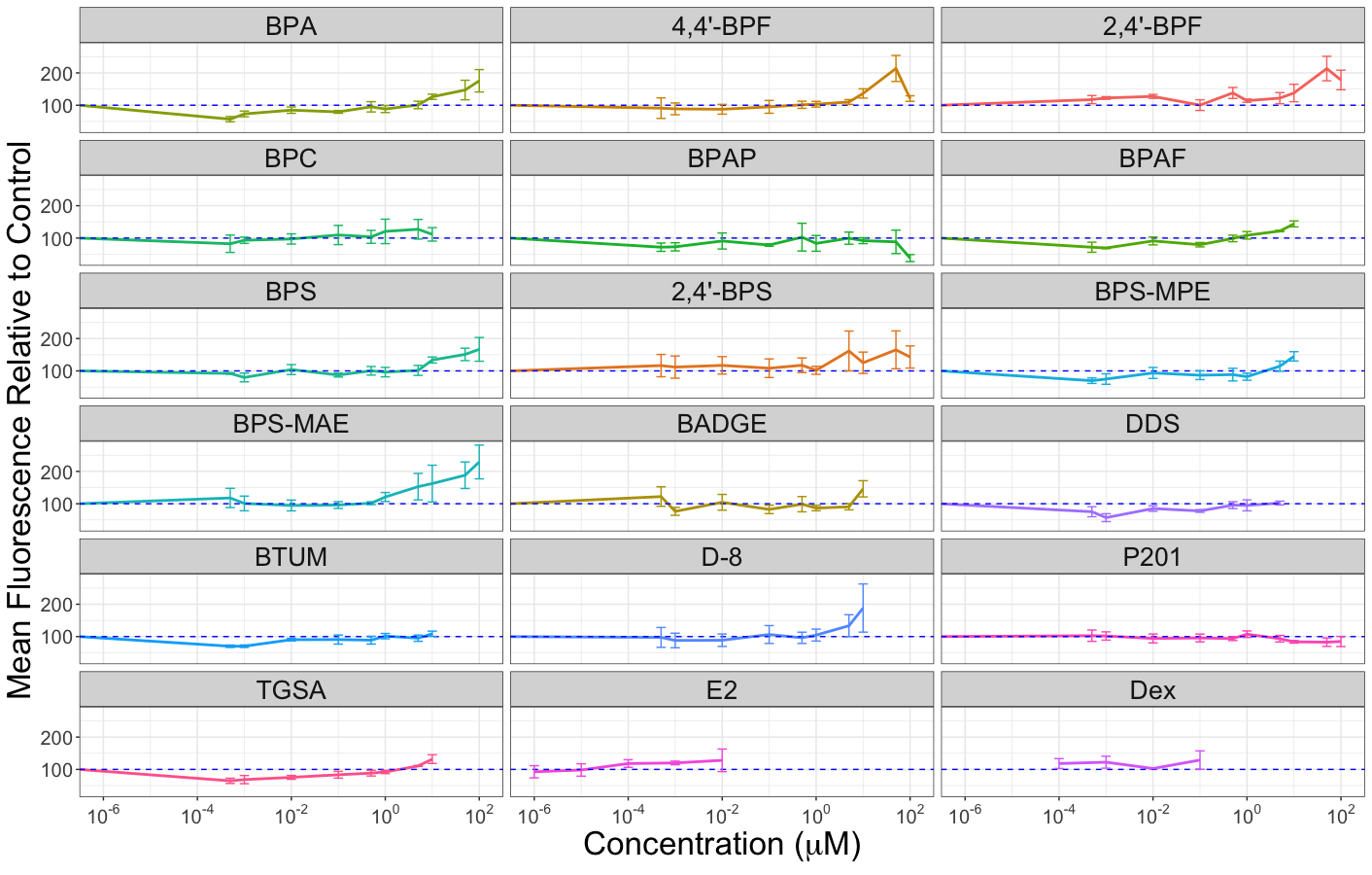


**Supplementary Figure S1**: Cell viability using a CellTiter-Blue Cell Viability Assay. MCF-7 cells (n = 3 – 4) were exposed to BPA alternative chemicals at a range of concentrations (0.0005 - 100 µM) for 48 hours and are compared to their respective DMSO (0.1%) control samples. First row of chemicals from left to right: BPA, 4,4’-BPF, 2,4’-BPF. Second row: BPC, BPAP, BPAF. Third row: BPS, 2,4’-BPS, BPS-MPE. Fourth Row: BPS-MAE, BADGE, DDS. Fifth row: BTUM, D-8, P201. Fifth row: TGSA, E2, Dex. Blue horizontal dotted line denotes 100% cell viability relative to controls. There were no significant declines in viability relative to controls.

**
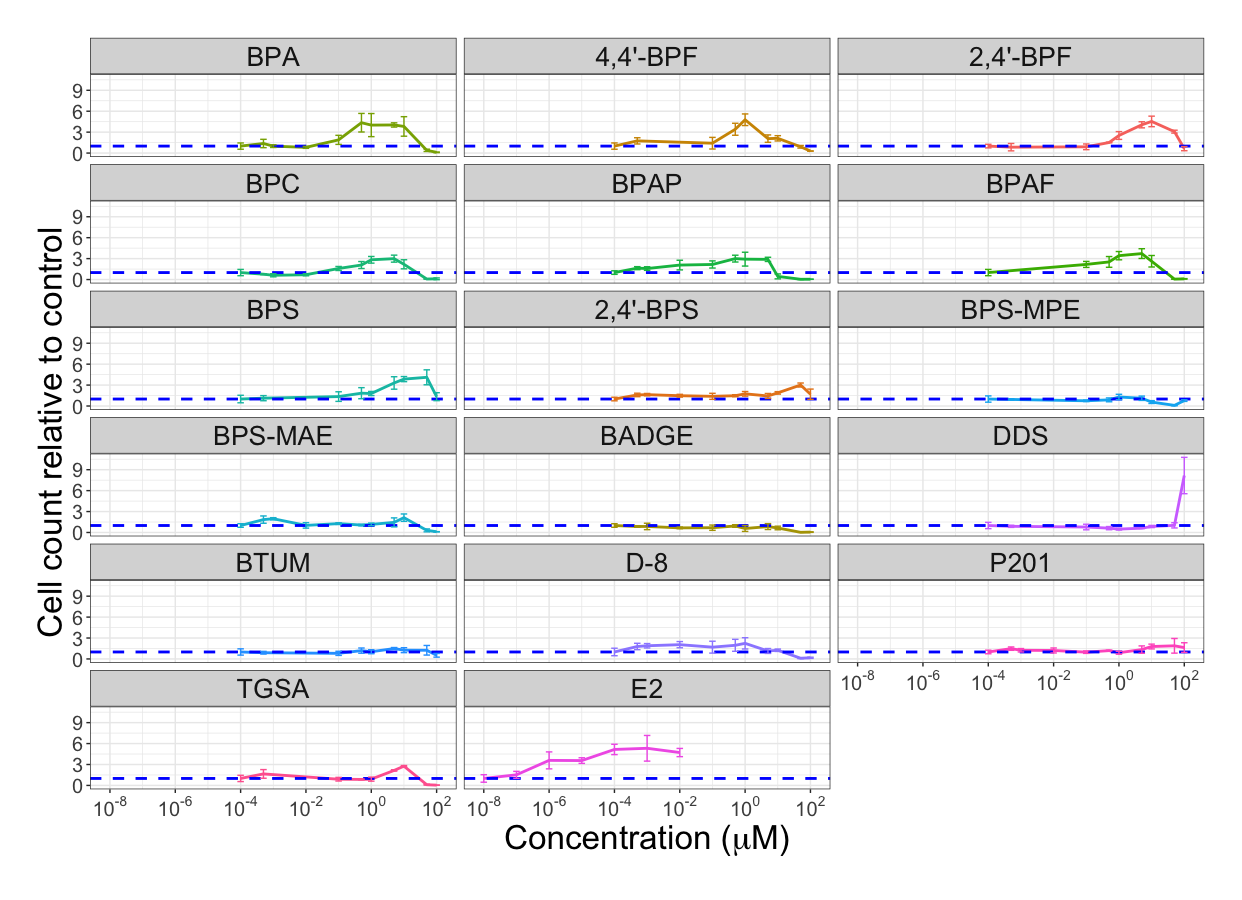
**

**Supplementary Figure S2**: Cell proliferation assay. MCF-7 cells (n = 3 – 4) were exposed to BPA alternative chemicals at a range of concentrations (0.0001 - 100 µM) for 7 days and are compared to their respective DMSO (0.1%) control samples. First row of chemicals from left to right: BPA, 4,4’-BPF, 2,4’-BPF. Second row: BPC, BPAP, BPAF. Third row: BPS, 2,4’-BPS, BPS-MPE. Fourth Row: BPS-MAE, BADGE, DDS. Fifth row: BTUM, D-8, P201. Fifth row: TGSA, E2. Blue horizontal dotted line denotes 100% cell proliferation relative to controls. The following chemicals increased proliferation relative to controls: BPA (0.0005, 0.1, 0.5, 1, 5, 10 µM), 4,4’-BPF (0.0005, 0.1, 0.5, 1, 5, 10 µM), 2,4’-BPF (0.5 – 50 µM), BPC (0.1 – 10 µM), BPAP (0.0005 – 5 µM), BPAF (0.1 – 10 µM), BPS (0.0005, 0.1, 0.5, 1, 5, 10, 50, 100 µM), 2,4’-BPS (0.0005 – 100 µM), BPS-MPE (1, 5 µM), BPS-MAE (0.0005 – 10 µM), DDS (50, 100 µM), BTUM (0.5 – 50 µM), D-8 (0.0005 – 10 µM), P201 (0.0005, 0.001, 0.01, 0.5, 5, 10, 50, 100), TGSA (0.0005, 0.5, 10 µM), E2 (0.0000001 – 0.01 µM). The following chemicals decreased cell proliferation below 50% of solvent control: BPA (50, 100 µM), 4,4’-BPF (100 µM), BPAP (10, 50, 100 µM), BPS-MAE (50, 100 µM), 2,4’-BPF (100 µM).

| Chemical | HDACi | NFkB | HSF1 | Nrf2 | MTF1 | TGx-DDI |
| --- | --- | --- | --- | --- | --- | --- |
| 2,4'-BPF |  | 50 |  | 50, 100 |  |  |
| 2,4'-BPS |  | 100 |  |  |  |  |
| 4,4'-BPF | 5, 10, 50, 100 |  |  | 100 |  | 10, 50 |
| BADGE |  |  |  |  | 0.1, 0.5, 1 |  |
| BPA | 5, 10, 50 |  |  | 100 | 1, 10 | 10 |
|  |  |  |  |  | 100 |  |
| BPAF | 1 |  |  |  | 1, 5 | 0.1 |
| BPAP | 5, 10, 50 | 100 | 50, 100 | 50, 100 | 50, 100 | 100 |
| BPC | 5, 10 |  |  |  | 5, 10 |  |
| BPS | 5 | 100 |  |  | 0.001 |  |
| BPS-MAE |  |  |  | 100 | 100 |  |
| BPS-MPE |  |  |  |  |  |  |
| BTUM |  |  |  |  | 5 |  |
| D-8 |  |  |  |  |  |  |
| DDS |  |  |  |  |  |  |
| P201 |  | 100 |  |  | 0.01, 0.1, 1, 10, 100 |  |
| TGSA |  |  |  |  |  |  |

**Supplementary Table S1**: Summary of stress response biomarkers of MCF-7 cells (n = 3 – 4) exposed to BPA alternative chemicals at a range of concentrations (0.0005 - 100 µM) for 48 hours. Biomarkers were activated (orange) or inhibited (blue) based on thresholds for significance −log(p-value) ≥4 or ≤−4.

| Chemical | 0.0005 µM | 0.001 µM | 0.01 µM | 0.1 µM | 0.5 µM | 1 µM | 5 µM | 10 µM | 50 µM | 100 µM |
| --- | --- | --- | --- | --- | --- | --- | --- | --- | --- | --- |
| 2,4'-BPF |  |  |  |  |  |  |  |  | 2 stress BMs | ↓proliferation |
| 2,4'-BPS |  |  |  |  |  |  |  |  |  |  |
| 4,4'-BPF |  |  |  |  |  |  |  |  |  | ↓proliferation |
| BADGE |  |  |  |  |  |  |  |  | precipitated | |
| BPA |  | TE | TE |  |  |  |  |  | ↓proliferation | 2 BMs & ↓ prolif |
| BPAF |  |  |  |  |  |  |  |  | precipitated | |
| BPAP |  |  |  |  |  |  |  | ↓proliferation | 2 BMs & ↓ prolif | |
| BPC |  |  |  |  |  |  |  |  | precipitated | |
| BPS |  |  |  |  |  |  |  |  |  |  |
| BPS-MAE |  |  |  |  |  |  |  |  | ↓proliferation | 2 BMs & ↓ prolif |
| BPS-MPE |  |  |  |  |  |  |  |  | precipitated | |
| BTUM |  |  |  |  |  |  |  |  | precipitated | |
| D-8 |  |  |  |  |  |  |  |  | precipitated | |
| DDS |  |  |  |  |  |  |  | precipitated | | |
| P201 |  |  |  |  |  |  |  |  |  | 2 stress BMs |
| TGSA |  |  |  |  |  |  |  |  | precipitated | |

**Supplementary Table S2:** Summary of concentrations removed from analysis. Samples were removed due to technical error (TE), visible precipitation (green), activating two or more stress response biomarkers (red; see Supplementary File S3), significantly decreasing cell proliferation (yellow; see Supplementary Figure S2), or both activating biomarkers and decreasing cell proliferation (orange).
